# Diverse Effects of Combinations of Maternal-Neonatal VDR Polymorphisms and 25-Hydroxyvitamin D Concentrations on Neonatal Birth Anthropometry: Functional Phenocopy Variability Dependence, Highlights the Need for Targeted Maternal 25-Hydroxyvitamin D Cut-Offs during Pregnancy

**DOI:** 10.3390/nu13020443

**Published:** 2021-01-29

**Authors:** Spyridon N. Karras, Erdinç Dursun, Merve Alaylıoğlu, Duygu Gezen-Ak, Cedric Annweiler, Dimitrios Skoutas, Dimosthenis Evangelidis, Dimitrios Kiortsis

**Affiliations:** 1 National Scholarship Foundation, 55535 Thessaloniki, Greece; 2 Brain and Neurodegenerative Disorders Research Laboratories, Department of Medical Biology, Cerrahpasa Faculty of Medicine, Istanbul University-Cerrahpasa, 34381 Istanbul, Turkey; erdincdu@gmail.com (E.D.); mariabioanalysis@yahoo.gr (M.A.); duygugezenak@gmail.com (D.G.-A.); 3 Department of Neuroscience, Institute of Neurological Sciences, Istanbul University-Cerrahpasa, 34381 Istanbul, Turkey; 4 Division of Geriatric Medicine, Department of Neuroscience, Angers University Hospital, 49035 Angers, France; ceannweiler@chu-angers.fr; 5 Robarts Research Institute, Department of Medical Biophysics, Schulich School of Medicine and Dentistry, The University of Western Ontario, London, ON N6A 3K7, Canada; 6 Sarafianos General Private Hospital, 54631 Thessaloniki, Greece; skoutasd@otenet.gr; 7 Εpsom and St Helier University Hospital NHS, London SM5 1AA, UK; dimos@doctors.org.uk; 8 Department of Nuclear Medicine, University of Ioannina, 45110 Ioannina, Greece; dkiorts@uoi.gr

**Keywords:** vitamin D, pregnancy, neonatal health, polymorphism, birth anthropometry

## Abstract

Vitamin D receptor (VDR) polymorphisms have been associated with a plethora of adverse pregnancy and offspring outcomes. The aim of this study was to evaluate the combined effect of maternal and neonatal VDR polymorphisms (ApaI, TaqI, BsmI, FokI, Tru9I) and different maternal and neonatal 25(OH)D cut-offs on neonatal birth anthropometry. This cross-sectional study included data and samples from a cohort of mother–child pairs at birth. A detailed neonatal anthropometry analysis at birth was also conducted. Different 25(OH)D cut-offs for neonates and mothers were included, according to their vitamin D status at birth: for neonates, cut-offs of [25(OH)D ≤ 25 and > 25 nmol/L] and [25(OH)D ≤ 50 nmol/L] were adopted, whereas for mothers, a 25(OH)D cut-off of [25(OH)D ≤ 50 and > 50 nmol/L)] was investigated. Following this classification, maternal and neonatal VDR polymorphisms were evaluated to investigate the potential different effects of different neonatal and maternal 25(OH)D cut-offs on neonatal birth anthropometry. A total of 69 maternal-neonatal dyads were included in final analysis. Weight, neck rump length, chest circumference, abdominal circumference, abdominal circumference (iliac), high thigh circumference, middle thigh circumference, lower arm radial circumference, and lower leg calf circumference of neonates who had the TAQl SNP TT genotype and maternal 25(OH)D < 50 nmol/L were significantly higher than that of neonates who had the Tt or tt genotypes (*p* = 0.001, H*g* = 1.341, *p* = 0.036, H*g* = 0.976, *p* = 0.004, H*g* = 1.381, *p* = 0.001, H*g* = 1.554, *p* = 0.001, H*g* = 1.351, *p* = 0.028, H*g* = 0.918, *p* = 0.008, H*g* = 1.090, *p* = 0.002, H*g* = 1.217, and *p* = 0.020, H*g* = 1.263, respectively). Skin fold high anterior was significantly lower in neonates who had the BSMI SNP BB genotype compared to that of neonates with Bb or bb genotypes (*p* = 0.041, H*g* = 0.950), whereas neck rump length was significantly higher in neonates who had the FOKI SNP FF genotype compared to that of neonates who had Ff or ff genotypes (*p* = 0.042, H*g* = 1.228). Regarding neonatal VDR polymorphisms and cut-offs, the abdominal circumference (cm) of neonates who had the TAQI SNP TT genotype and 25(OH)D < 25 nmol/L were significantly higher than that of neonates who had the Tt or tt genotypes (*p* = 0.038, H*g* = 1.138). In conclusion, these results indicate that the maternal TAQI VDR polymorphism significantly affected neonatal birth anthropometry when maternal 25(OH) concentrations were <50 nmol/L, but not for a higher cut-off of >50 nmol/L, whereas this effect is minimally evident in the presence of neonatal TAQI polymorphism with neonatal 25(OH)D values <25 nmol/L. The implication of these findings could be incorporated in daily clinical practice by targeting a maternal 25(OH)D cut-off >50 nmol/L, which could be protective against any effect of genetic VDR variance polymorphism on birth anthropometry.

## 1. Introduction

International nutritional recommendations during pregnancy comprise a fundamental guide for the optimal fetal development. Although there is almost universal agreement regarding macronutrient and folic requirements as well as the monitoring of weight gain during pregnancy, there are significant controversies regarding supplementation with specific biological molecules including vitamins [1].

A plethora of observational trials indicate maternal hypovitaminosis D during pregnancy as a significant risk factor for the development of adverse pregnancy outcomes and impairment of future offspring metabolic health [2]. Despite the wide inconsistency in available randomized trials, it is considered that vitamin D supplementation during pregnancy might reduce the risk of low birth weight, gestational diabetes, and preeclampsia [2,3]. In this regard, a sufficient maternal vitamin D profile during pregnancy is a critical component for the development of optimal neonatal vitamin D status at birth and during early infancy, since maternal 25-hydroxy-vitamin D [25(OH)D] comprises the main pool of vitamin D for the fetus [3] and serum fetal (cord blood) 25(OH)D concentrations correlate strongly with maternal 25(OH)D concentrations [4,5]. Τhere is wide controversy, about the definition of maternal vitamin D deficiency during pregnancy worldwide, especially regarding the optimal thresholds of maternal 25(OH)D concentrations (≥50 nmol/L vs. ≥75 nmol/L) [6,7]. On the other hand, different criteria are used to define the optimal neonatal vitamin D status (sufficiency >50 nmol/L, insufficiency 30–50 nmol/L, deficiency <30 nmol/L) [8].

In addition to this ongoing scientific argument, several parameters that could affect the daily clinical interpretation of available results, beyond maternal and neonatal 25(OH)D cut-offs including ultraviolet B (UVB) variations, country-specific dietary patterns, and public health policies are largely misinterpreted or ignored [8,9]. The potential influence of the specific genetic background of each individual for decreasing pregnancy complications and optimizing neonatal health has also been the objective of several previous observational studies, which mainly focused on the specific sequence variants of the vitamin D receptor (VDR). Specific maternal VDR polymorphisms have been associated with adverse pregnancy and offspring outcomes [10,11,12] and could demonstrate either detrimental or protective effects [10,11,12,13] on the development of maternal and neonatal hypovitaminosis D and other outcomes.

The extent of the potential effects of specific neonatal VDR polymorphisms related to neonatal 25(OH)D by adopting international cut-offs has not been fully elucidated so far [2,7]. By taking into account that maternal and neonatal vitamin D status at birth are dynamic parameters, in order to understand the mechanistic basis by which a polymorphism is associated with a particular pregnancy or offspring outcome, it is necessary to know whether that polymorphism is functional in different states of vitamin D equilibrium. In this regard, specific VDR polymorphisms have been associated with a decrease in birth weight and neonatal skin folds at birth [14] and increased risk for small for gestational age births in black and white women [15,16]. In addition, placental genetic variations in vitamin D metabolism were also associated through a sex-specific pattern with birth weight [17]. A recent meta-analysis of available results reported that birth weight and other anthropometric neonatal outcomes are affected by specific patterns of VDR polymorphisms [18]. Moreover, maternal genetic variations in GC, the gene encoding vitamin-D binding protein, have also been reported to affect the relationships between the maternal and cord-blood concentrations of 25(OH)D and birth weight [19]. However, robust evidence of such an association is currently unavailable, given that various studies have presented significant heterogeneity in terms of maternal and neonatal criteria for vitamin D status, study design, sample size, and racial descent of participants.

It has been hypothesized [20] that specific sequence VDR variants exert variable degrees of functionality associated with a specific neonatal outcome including birth height, weight, and additional birth anthropometry parameters, according to different cut-offs and available maternal and neonatal VDR polymorphisms. Moreover, a combined clinical (in terms of different maternal/neonatal 25(OH) D cut-offs) and VDR genotype association study focusing on a specific outcome could be mechanistically proven to be more productive than a study of individual polymorphisms or genome-wide associations of polymorphisms of unknown function. The aim of this study was to evaluate the combined effect of maternal and neonatal VDR polymorphisms (ApaI, TaqI, BsmI, FokI, Tru9I) and different maternal and neonatal 25(OH) D cut-offs on neonatal birth anthropometry.

## 2. Methods

### 2.1. Inclusion and Exclusion Criteria

This study included data and samples from a cohort of mother–child pairs at birth that have been previously described [3]. Pregnant women on regular follow-up were recruited from the Maternity Unit of the 1st Department of Obstetrics and Gynecology, Aristotle University, Thessaloniki, Greece (latitude 40°N). All women were fair skinned. The inclusion criterion was full-term pregnancy (gestational week 37–42). Maternal exclusion criteria were primary hyperparathyroidism, secondary osteoporosis, heavy alcohol use (≥7 alcohol units per week or ≥6 units at any time during pregnancy), hyperthyroidism, nephritic syndrome, inflammatory bowel disease, rheumatoid arthritis, osteomalacia, obesity [body mass index (BMI) > 30 kg/m^2^], gestational diabetes, preexisting diabetes mellitus, and use of medications affecting calcium (Ca) or vitamin D status (e.g., corticosteroids) including vitamin D supplements. Neonatal exclusion criteria were being small-for-gestational age (SGA) and presence of severe congenital anomalies. Informed consent was obtained from all mothers. The study was conducted from January 2018 to September 2018. The protocol received approval from the Bioethics Committee of the Aristotle University of Thessaloniki, Greece (approval number 1/19-12-2011).

### 2.2. Biochemical and Hormonal Assays

Blood samples were obtained from mothers by antecubital venipuncture 30–60 min before delivery. Umbilical cord blood was collected immediately after clamping from the umbilical vein. Serum and umbilical cord specimens were stored at −20 °C prior to analysis for the following parameters: Ca, phosphorus (P), parathyroid hormone (PTH), and 25(OH)D. Serum Ca and P determinations were performed using the Cobas INTEGRA clinical chemistry system (D-68298; Roche Diagnostics, Mannheim, Germany). The inter- and intra-assay coefficients of variation (CVs) were 1.0% and 3.5% for Ca, and 1.3% and 2.5% for P, respectively. PTH determinations were performed using the electro-chemiluminescence immunoassay ECLIA (Roche Diagnostics GmbA, Mannheim, Germany). Reference range for PTH was 15–65 pg/mL, functional sensitivity of 6.0 pg/mL, within-run precision of 0.6–2.8%, and total precision of 1.6–3.4%. Concentrations of 25(OH)D were determined using novel assay, liquid chromatography-tandem mass spectrometry (LC-MS/MS) with lower limits of quantification (LLOQ) of 25(OH)D (0.5 ng/mL). Briefly, the assay involves analyte purification using liquid–liquid extraction followed by chromatographical separation using a chiral column in tandem with a rapid resolution microbore column. Full method validation parameters have been previously reported [21,22].

### 2.3. Demographic and Anthropometric Data

At enrollment, demographic and social characteristics were recorded. Maternal pre-pregnancy BMI was either normal (18–25 kg/m^2^) or overweight (25–30 kg/m^2^). We collected maternal, infant, and labor data from the medical records, umbilical cord blood samples at the time of delivery, and stored aliquots of plasma and serum at −70 °C, until assays were performed. We also evaluated neonatal anthropometry at birth. All neonatal anthropometric measurements were performed by the same trained nurse between 12 and 72 h of age according to standard techniques [23]. The following measurements were recorded: birth weight, height, neck-rump length, upper arm, femur, and knee-heel lengths; head, chest, abdominal, upper arm and middle thigh circumferences, and anterior chest and abdominal skinfold thickness. Birth weight of the neonates was obtained naked on regularly calibrated scales on a calibrated infant scale that was verified as accurate with a certified weight (Troemner, Thorofare, NJ, USA). Knee–heel length was measured with a hand-held BK5 infant knemometer (Force Technology, Brondby, Denmark). Instrument software calculated the mean of 10 sequential readings and generated a printed report of all readings and the calculated mean. We also measured the neonatal height to the nearest millimeter using an Ellard newborn lengthboard (Ellard Instrumentation Ltd., Seattle, WA, USA). Abdominal, upper arm and middle thigh head, mid-upper arm, and maximal head circumferences were measured using a plastic encircling tape (Child Growth Foundation, London, UK). Abdominal skin fold was measured using Holtain calipers (Holtain, Crymych, UK).

### 2.4. Neonatal and Maternal Vitamin D Status Cut-Offs and Combined VDR Polymorphisms Evaluation

Different 25(OH)D cut-offs for neonates and mothers were included, according to their vitamin D status at birth: for neonates, cut-offs of [25(OH)D ≤25 and >25 nmol/L] and [25(OH)D ≤50] [7] were adopted, whereas for mothers, a 25(OH)D cut-off of [25(OH)D ≤50 and >50 nmol/L)] was investigated [24]. Following this classification, maternal and neonatal VDR polymorphisms were assessed at birth to investigate the potential different effects of different neonatal and maternal 25(OH)D cut-offs on neonatal birth anthropometry.

### 2.5. VDR Analysis

DNA was isolated from peripheral blood samples by a QIAamp DNA Blood Mini Kit (Cat. no. 51304, QIAGEN), according to the manufacturer’s protocol. In order to determine the genotypes of rs7975232 (ApaI), rs7731236 (TaqI), rs757343 (Tru9I), and rs1544410 (BsmI) SNPs within the *VDR* gene, polymerase chain reaction (PCR) and restriction fragment length polymorphism (RFLP) methods were performed as previously described [25]. The real-time PCR (RT-PCR) method was used for determining genotypes of rs2228570 (FokI) SNP by using the Simple Probe (LightSNiP, TibMolBiol, Berlin, Germany) and LightCycler Fast Start DNA Master HybProbe Kit (Cat. no. 12239272001, Roche) with a LightCycler 480 Instrument II (Roche). Melting curve analysis was performed for genotyping as previously described [26].

Each SNP allele was named after as follows: for rs7731236 (TaqI), “t” represents C, “T” represents T nucleotide; for rs7975232 (ApaI), “a” represents C, “A” represents A nucleotide; for rs757343 (Tru9I), “u” represents A, “U” represents G nucleotide; for rs1544410 (BsmI), “b” represents G, “B” represents A nucleotide, and for rs2228570 (FokI), “f” represents T, and “F” represents C nucleotide.

### 2.6. UVB Measurements

UVB radiation includes wavelengths from 280 to 320 nm. UVB data for the broad geographical region of Thessaloniki, Greece were collected at the Laboratory of Atmospheric Physics, School of Physics, Aristotle University of Thessaloniki.

The daily integral of vitamin D effective UVB radiation (09:00 to 16:00 local time) was used as the most representative parameter for UVB exposure. These hours were selected as indicative, since they are related to the beginning and the end of the working period for the majority of the Greek population. Individual sunlight exposure was recorded for each participant during that period. Finally, mean UVB exposure during the previous 45 days (daily integral) before blood sample collection (estimated mean half-life of vitamin D) was calculated for each participant.

### 2.7. Statistical Analysis

Given that there was no comparison group, the distributions of genotypes of SNPs within the maternal and neonatal VDR genes were given as frequency data. Mean birth neonatal anthropometry data including height (cm), weight (g), head circ/ce (cm), neck rump length (cm), chest circ/ce (cm), abdominal circ/ce (cm), abdominal circ/ce iliac (cm), skin fold abdominal (cm), skin fold high anterior (cm), high thigh circ/ce (cm), middle thigh circ/ce (cm), upper arm length (cm), lower arm radial circ/ce (cm), lower leg calf circ/ce (cm), femur length (cm), and knee-heel length (cm) values of minor allele carriers and homozygote major allele carriers in groups were compared with *T*-Tests. If the Levene’s test for equality of variances is *p* > 0.05, then equal variances assumed Sig (2-tailed) *p* values of *T*-Tests were given. If the Levene’s test for equality of variances is *p* < 0.05, then the equal variances not assumed Sig (2-tailed) *p* values of *T*-Tests were given. The data and *p* values adjusted for maternal and paternal height (cm), UVB radiation, BMI pre-pregnancy (kg/m^2^), BMI terminal (kg/m^2^), and weeks of gestation by one-way analysis of covariance (ANCOVA). Corrected effect size was calculated with Hedge’s g (H*g*) where 0.2 is suggested as a small effect size, 0.5 is the medium effect, and 0.8 is a larger effect [27]. Post-hoc power analysis was performed for significant outcomes. All data were presented as the means ± SD in the text and figure legends. The tests were performed in groups by stratifying data for maternal or neonatal 25OHD level cutoff values. Statistical analyses were performed by SPSS 24.0 software(IBM, Armonk, New York, NY, USA).

## 3. Results

Seventy mother–neonate pairs were included in the study. Given four neonates had missing birth neonatal anthropometry data, they were excluded from related analysis. The demographic and laboratory data of mothers and neonates are presented in Table 1. VDR single nucleotide polymorphisms (SNPs) and the genotype distributions of mothers and neonates are presented in Table 2.

### 3.1. Birth Neonatal Anthropometry (Neonatal Cut-Offs at Birth >50 nmol/L and <25 and >25 nmol/L) according to Neonatal VDR Polymorphisms

Birth neonatal anthropometry was investigated in neonates whose 25(OH)D at birth was <25 and >25 nmol/L, respectively, and compared according to neonatal VDR polymorphisms. After adjustments, the abdominal circumference (cm) of neonates who had the TAQI SNP TT genotype and 25(OH)D < 25 nmol/L were significantly higher than that of neonates who had Tt or tt genotypes (*p* = 0.038, H*g* = 1.138) (Table 3), whereas for neonates with 25(OH)D >25 nmol/L, no significant difference was observed in any birth neonatal anthropometry (Table 4). There was no significant difference in any additional birth neonatal anthropometry parameter, which was investigated in neonates with 25(OH) D > 50 nmol/L, according to neonatal VDR polymorphisms (Table 5).

### 3.2. Birth Neonatal Anthropometry (Maternal Cut-Offs at Birth <50 and >50 nmol/L) According to Maternal VDR Polymorphisms

Birth neonatal anthropometry was investigated in neonates whose maternal 25(OH)D at birth was <50 and >50 nmol/L, respectively, and compared according to maternal VDR polymorphisms. After adjustments, weight, neck rump length, chest circumference, abdominal circumference, abdominal circumference (iliac), high thigh circumference, middle thigh circumference, lower arm radial circumference, and lower leg calf circumference of neonates who had the TAQl SNP TT genotype and maternal 25(OH)D <50 nmol/L were significantly higher than that of neonates who had the Tt or tt genotypes (*p* = 0.001, H*g* = 1.341, *p* = 0.036, H*g* = 0.976, *p* = 0.004, H*g* = 1.381, *p* = 0.001, H*g* = 1.554, *p* = 0.001, H*g* = 1.351, *p* = 0.028, H*g* = 0.918, *p* = 0.008, H*g* = 1.090, *p* = 0.002, H*g* = 1.217, and *p* = 0.020, H*g* = 1.263; respectively), (Table 6). Skin fold high anterior was significantly lower in neonates who had the BSMI SNP BB genotype, compared to that of neonates with Bb or bb genotypes (*p* = 0.041, H*g* = 0.950) (Table 6), whereas neck rump length was significantly higher in neonates who had the FOKI SNP FF genotype compared to that of neonates who had Ff or ff genotypes (*p* = 0.042, H*g* = 1.228) (Table 6).

There was no significant difference in any additional birth neonatal anthropometry parameter, which was investigated in neonates whose maternal 25(OH)D concentration was >50 nmol/L when compared according to maternal VDR polymorphisms, except neonatal height. The height of the neonates with UU was significantly higher than the ones with Uu or uu (*p* = 0.032, H*g* = 0.444) (Table 7).

### 3.3. Birth Neonatal Anthropometry (Maternal Cut-Off at Birth <75 nmol/L) According to Neonatal VDR Polymorphisms

Birth neonatal anthropometry was also investigated in neonates whose maternal 25(OH)D was <75 nmol/L and compared according to neonatal VDR polymorphisms. After adjustments, the lower arm radial circumference of neonates who had the APAl SNP AA genotype was significantly lower than that of neonates who had Aa or aa genotypes (*p* = 0.043, H*g* = 0.966) (Table 8), whereas no other significant differences were evident (Table 8).

## 4. Discussion

Τhis study aimed to evaluate the combined effects of maternal and neonatal VDR polymorphisms (ApaI, TaqI, BsmI, FokI, Tru9I) and different maternal and neonatal 25(OH) D cut-offs on neonatal birth anthropometry at birth including a population from a sunny Mediterranean area in Northern Greece. Results from this maternal–neonatal pair cohort indicate that: (i) the maternal TAQI VDR polymorphism significantly affects neonatal birth anthropometry when maternal 25(OH)D concentrations are <50 nmol/L, but not for a higher cut-off of >50 nmol/L, (ii) neonatal VDR polymorphisms combined with neonatal 25(OH)D > 25 nmol/L have negligible effects on birth anthropometry, whereas this combination exerts a minimal effect, in the presence of neonatal TAQI polymorphism with neonatal 25(OH)D values <25 nmol/L, and (iii) FOKI and BSMI maternal VDR polymorphisms demonstrate minimal—out of a consistent pattern—effects on skin fold and neck-rump length, which warrant further investigation in future studies with larger samples from mothers and neonates.

These findings are the first to be reported on the combined effects of maternal and neonatal VDR polymorphisms and respective 25(OH)D cut-offs on neonatal birth anthropometry from this region. Moreover, these findings indicate that there are variable degrees of VDR polymorphism functionality depending on maternal and neonatal 25(OH)D concentrations, which result in different anthropometric patterns at birth. In the daily clinical setting, these findings also identify different ‘’safe’’ maternal 25(OH)D cut-offs (>50 nmol/L for maternal and >25 nmol/L for neonatal vitamin D status), whose attainment practically prevents genetic functional VDR influences on a given neonatal outcome. This is the first mechanistic study of this kind, which combines both aspects of vitamin D physiology, fluctuating concentrations of 25(OH) D, and common VDR polymorphisms with a discourse on the specific neonatal outcome.

Although associations between VDR polymorphisms with a plethora of adverse pregnancy outcomes such as preterm birth and SGA neonates [10,11,12,13,14,15,16,17,18] have been suggested, evidence is still inconclusive, primarily due to the lack of a pathophysiological connection of individual vitamin D status and functionality of VDR polymorphisms. The most commonly investigated polymorphisms included were the BsmI (rs1544410), ApaI (rs7975232), FokI (rs2228570), and TaqI (rs731236) polymorphisms, while TaqI and FokI consisted of a single base change (A to G and G to A in exons 9 and 2, respectively), and BsmI and ApaI were located in the last intron of the sequence and resulted from a single base change (G to A and A to C, respectively). However, results in the field are highly inconsistent, mainly due to the absence of incorporated standardized thresholds of vitamin D status in the initial study design, which could enable a universal stratification of mothers and neonates. The racial diversity of included populations might also contribute to the inconsistency of the results, underlying the importance of regionally-derived data in the implementation of national health policies [6,28,29].

We have previously highlighted the importance of population-specific genetic profiling in understanding vitamin D deficiency among neonates and their mothers and the protective effect of the maternal Fokl FF genotype against the development of neonatal vitamin D deficiency [25(OH)D <30 nmol/L] [13]. However, there was a lack of simultaneous assessment of both maternal and neonatal VDR polymorphisms in one snapshot, with different 25(OH)D thresholds, oriented toward a detailed evaluation of birth anthropometry as a method of crude estimation of neonatal adiposity [23], which could identify an adverse metabolic offspring profile in later adult life [30].

We included a maternal cut-off of 50 mol/l, but not one of 75 nmol/L, since our cohort did not include women with higher 25(OH)D concentrations, since none of them was supplemented. On the other hand, we considered that including a maternal cut-off of 25 nmol/L would be far from the widely adopted international recommendations for maternal values during pregnancy [2,5,18]. Our goal was to explore vitamin D status and VDR polymorphism interactions in the most common equilibrium pattern observed in non-supplemented women from our region. We followed the same rationale for neonates by including lower cut-offs of 25(OH)D, based on previous observations on maternal–neonatal vitamin D equilibrium at birth [3,4]. Future studies with higher maternal and neonatal cut-offs could be useful in exploring the broader spectrum of maternal–neonatal interactions in this setting.

Mechanistic pathways between VDR expression and offspring outcomes remain largely unclear. Apart from its classical intracellular pathways, which allows the ligand-bound VDR to form heterodimers with nuclear retinoid X receptor (RXR) and recruit co-factors to modulate gene transcription [31], vitamin D can also exert rapid non-genomic effects, probably via VDR located within the plasma membrane [32,33]. However, the functional effects of VDR and its allelic variants on birth anthropometry have not been elucidated, until recently.

Interestingly, we observed that maternal and neonatal TaqI polymorphism is a significant modulator of neonatal birth anthropometry when maternal and neonatal values are in a range of <50 nmol/L and <25 nmol/L, respectively. Results about the effect of VDR polymorphisms including the TaqI polymorphism on neonatal birth anthropometry are currently inconclusive: Swamy and colleagues [16] prospectively evaluated the effect of 38 VDR polymorphisms on several birth outcomes on 615 pregnant women including birth weight. A total of eight out of 38 SNPs examined significantly affected birth weight in black but not in white women, indicating a biologically plausible association that could depend on ethnicity, providing a partial explanation for the observed racial disparity in several pregnancy outcomes.

In a previous study including participants of Caucasian origin, boys with the BB genotype were shorter at birth and grew less from birth to the age of 16.9 than their Bb and bb counterparts. A prediction model including parental height, birth height, birth weight, and VDR alleles could predict up to 39% of the total variation in adult height [34]. Similarly, in a maternal–neonatal cohort from Australia [14], neonates of vitamin D deficient mothers had lower birth weight with FF or Ff, but not ff genotype, whereas thicker subscapular and suprailiac skinfolds with ff, but not the FF or Ff genotype. Placental genetic variations in vitamin D metabolism through investigation of five vitamin D metabolism genes (CUBN, LRP2, VDR, GC, and CYP2R1) was also associated through a sex-specific pattern with birthweight, but not with other neonatal outcomes [17]. In our study, although BSMI SNP BB and FOKI SNP FF genotypes were associated with anterior skin fold and neck rump length and a maternal 25(OH)D cut-off <50 nmol/L, we considered that these findings did not establish a solid biological effect that could identify a genetic variation pattern, as evident for TaqI, where a plethora of birth anthropometry parameters were uniformly affected.

Regarding the effect of TaqI polymorphism on neonatal anthropometry, Barchitta et al. [18] reported that birth weight increased with an increasing number of mutated alleles, concluding that a beneficial effect of TaqI polymorphism in this regard could not be ruled out. However, these results should be interpreted with caution due to the high heterogeneity in design and outcomes, sample size, ethnicity, geographical diversity, sun exposure, dietary calcium and vitamin D intake, and maternal habits [35,36,37]. This study has certain limitations. First, the sample size was small and not powered to detect additional differences in other maternal-neonatal cut-offs, but it was sufficiently powered to show significant differences regarding the main aim of the study. Second, the cross-sectional design of the study could not prove a causal relationship. Third, all women were Caucasian, so our results cannot be safely generalized to other ethnicities, known to differ at least in the frequency of VDR polymorphisms, indicating that further similar studies from other regions could be useful, in order to elucidate the full extent of the ethnic VDR variation effect in neonatal outcomes. Finally, gender influences on anthropometric parameters in association with specific VDR polymorphisms have not been assessed due to the limited number of neonates included in the study. Therefore, the hypothesis of a gender-specific effect requires future investigation in studies with larger samples.

In conclusion, these results indicate that maternal TAQI VDR polymorphism significantly affects neonatal birth anthropometry, when maternal 25(OH) concentrations are <50 nmol/L, but not for a higher cut-off of >50 nmol/L, whereas this effect is minimally evident in the presence of neonatal TAQI polymorphism with neonatal 25(OH)D values <25 nmol/L.

No other effects of VDR common polymorphisms were evident using specific maternal and neonatal cut-offs. The implications of these findings could be incorporated in daily clinical practice by targeting a maternal 25(OH)D cut-off > 50 nmol/L, which could be protective against any potential effect of genetic VDR polymorphism variances on neonatal birth anthropometry.

## Figures and Tables

**Table 1:** Maternal and neonatal demographic and anthropometric characteristics.

***Maternal***	
Number (n)	66
Age (years)	31.92 ± 6.08
Height (cm)	164.85 ± 5.47
Weight; pre-pregnancy (kg)	67.56 ± 14.54
Weight; term (kg)	85.43 ± 14.30
BMI; pre-pregnancy (kg/m^2^)	24.91 ± 4.81
BMI; term (kg/m^2^)	29.62 ± 5.80
Weeks of gestation (n)	38.80 ± 1.56
Smoking [n (%)]	10 (0.14)
Alcohol consumption [n (%)]	8 (0.11)
Previous live births [n (%)]	26 (0.37)
Daily Calcium Supplementation [n (%)]	37 (0.56)
Daily Calcium Supplementation (mg)	423.07 ± 319.07
Paternal height	177.85 ± 6.14
***Neonatal***	
Number (n)	66
Gender; Males [n (%)]	38 (0.58)
Height (cm)	50.48 ± 1.96
Weight (g)	3292.12 ± 414.25
Head Circumference (cm)	34.40 ± 2.83
Neck rump length (cm)	17.66 ± 2.16
Chest Circumference (cm)	30.97 ± 1.97
Abdominal Circumference (cm)	28.11 ± 2.03
Abdominal Circumference iliac (cm)	25.94 ± 1.71
Skin fold; abdominal (cm)	2.95 ± 0.50
Upper Arm Circumference (cm)	9.74 ± 0.74
High thigh Circumference (cm)	15.41 ± 1.48
Middle thigh Circumference (cm)	13.36 ± 1.16
Upper Arm Length (cm)	13.65 ± 0.94
Lower Leg Calf Circumference (cm)	10.23 ± 0.83
Femur Length (cm)	9.94 ± 0.57
Knee-Heel Length (cm)	9.15 ± 0.62

Data are presented as mean ± standard deviation (SD) for continuous variables and frequencies [numbers (%)] for categorical variables. Abbreviations: BMI, body mass index.

**Table 2:** Vitamin D receptor single nucleotide polymorphisms genotype distributions of mothers and neonates.

SNP	APAI			TAQI			BSMI			FOKI			TRU9I		
Genotype	AA	Aa	aa	TT	Tt	tt	BB	Bb	bb	FF	Ff	ff	UU	Uu	uu
Maternal (n:%)	29 (0.41)	33 (0.47)	8 (0.12)	25 (0.36)	33 (0.47)	12 (0.17)	26 (0.37)	21 (0.30)	23 (0.33)	32 (0.46)	32 (0.46)	6 (0.08)	41 (0.59)	26 (0.37)	3 (0.04)
Neonatal (n:%)	23 (0.33)	39 (0.56)	8 (0.11)	27 (0.38)	32 (0.46)	11 (0.16)	19 (0.27)	27 (0.39)	24 (0.34)	34 (0.49)	31 (0.44)	5 (0.07)	46 (0.66)	22 (0.31)	2 (0.03)

**Table 3:** Birth neonatal anthropometry (neonatal vitamin D status at birth <25 nmol/L), according to neonatal VDR polymorphisms.

SNP	Genotype	N	Height (cm)	Weight (g)	Head Circ/ce (cm)	Neck Rump Length (cm)	Chest Circ/ce (cm)	Abdominal Circ/ce (cm)	Abdominal Circ/ce Iliac (cm)	Skin Fold Abdominal (cm)	Skin Fold High Anterior (cm)	High Thigh Circ/ce (cm)	Middle Thigh Circ/ce (cm)	Upper Arm Length (cm)	Lower Arm Radial Circ/ce (cm)	Lower Leg Calf Circ/ce (cm)	Femur Length (cm)	Knee-Heel Length (cm)
**APAl**	AA	9	49.50 ± 1.6	3261.11 ± 341	34.66 ± 0.7	17.82 ± 2.0	30.57 ± 2.5	27.95 ± 2.3	25.80 ± 1.6	2.71 ± 0.3	3.64 ± 0.5	15.24 ± 1.3	13.43 ± 1.5	13.44 ± 0.5	8.90 ± 0.7	10.33 ± 0.8	10.02 ± 0.4	9.40 ± 0.4
	Aa + aa	16	50.81 ± 1.9	3352.50 ± 372	35.06 ± 5.4	17.45 ± 2.0	31.20 ± 1.8	27.88 ± 1.6	26.01 ± 1.3	2.95 ± 0.2	4.00 ± 0.4	15.61 ± 1.1	13.55 ± 0.8	13.32 ± 0.7	8.96 ± 0.5	10.36 ± 0.6	9.93 ± 0.6	9.24 ± 0.6
	***p*-value**		0.1	0.55	0.83	0.67	0.48	0.92	0.72	0.06	0.09	0.47	0.8	0.69	0.81	0.9	0.73	0.52
**TAQl**	TT	8	50.62 ± 2.3	3523.75 ± 532	36.56 ± 7.5	18.95 ± 1.4	32.13 ± 2.3	29.10 ± 2.4	27.06 ± 1.9	3.02 ± 0.2	4.07 ± 0.3	16.32 ± 0.9	14.30 ± 1.3	13.51 ± 0.8	9.41 ± 0.8	10.76 ± 0.9	10.06 ± 0.7	9.48 ± 0.9
						(17.00 ± 1.2) *	(29.86 ± 1.3) *	(26.61 ± 1.3) *	(25.13 ± 0.7) *			(14.83 ± 1.0) *	(13.11 ± 0.9) *		(8.58 ± 0.3) *			
	Tt + tt	17	50.20 ± 1.7	3223.52 ± 190	34.14 ± 0.8	16.94 ± 1.9	30.43 ± 1.7	27.34 ± 1.7	25.41 ± 0.8	2.78 ± 0.3	3.77 ± 0.5	15.08 ± 1.1	13.13 ± 0.7	13.30 ± 0.6	8.71 ± 0.3	10.16 ± 0.4	9.92 ± 0.4	9.21 ± 0.3
						(17.48 ± 2.2) *	(31.37 ± 1.4) *	(28.09 ± 1.3) *	(26.00 ± 1.2) *			(15.76 ± 1.0) *	(13.52 ± 0.7) *		(9.00 ± 0.6) *			
	***p*-value**		0.62	0.16	0.39	**0.017**	**0.054**	**0.092**	**0.048**	0.073	0.17	**0.013**	**0.01**	0.49	**0.052**	0.11	0.58	0.43
	***adjusted p***					0.95 *	0.31 *	0.038 *	0.060 *			0.25 *	0.49 *		0.073 *			
	***Effect size***							***1.138 ^†^***										
	***Power***							***0.76 ^ϕ^***										
**BSMl**	BB	8	49.31 ± 1.6	3165.00 ± 195	34.56 ± 0.7	17.32 ± 1.5	29.95 ± 1.8	27.22 ± 0.9	25.28 ± 0.6	2.65 ± 0.3	3.57 ± 0.5	14.87 ± 0.8	12.97 ± 0.6	13.40 ± 0.6	8.86 ± 0.2	10.01 ± 0.4	9.96 ± 0.4	9.28 ± 0.2
										(2.93 ± 0.3) *	(3.93 ± 0.5) *							
	Bb + bb	17	50.82 ± 1.8	3392.35 ± 395	35.08 ± 5.2	17.71 ± 2.24	31.46 ± 2.0	28.22 ± 2.1	26.24 ± 1.6	2.96 ± 0.2	4.01 ± 0.4	15.76 ± 1.2	13.75 ± 1.1	13.35 ± 0.7	9.07 ± 0.7	10.47 ± 0.7	9.97 ± 0.6	9.30 ± 0.6
										(2.91 ± 0.3) *	(3.90 ± 0.4) *							
	***p*-value**		0.066	0.067	0.78	0.66	0.09	0.22	0.12	**0.014**	**0.04**	0.084	0.097	0.88	0.13	0.2	0.97	0.92
										0.80 *	0.80 *							
**FOKI**	FF	10	49.90 ± 1.5	3256.00 ± 307	34.25 ± 0.8	18.21 ± 1.5	30.94 ± 2.1	27.75 ± 2.2	25.89 ± 1.4	2.80 ± 0.2	3.70 ± 0.5	15.36 ± 1.2	13.63 ± 1.4	13.34 ± 0.5	8.78 ± 0.7	10.27 ± 0.8	10.05 ± 0.4	9.11 ± 0.6
	Ff + ff	15	50.63 ± 2.1	3362.00 ± 390	35.36 ± 5.5	17.17 ± 2.2	31.00 ± 2.1	28.01 ± 1.6	25.97 ± 1.5	2.90 ± 0.3	3.98 ± 0.4	15.56 ± 1.1	13.42 ± 0.8	13.38 ± 0.7	9.04 ± 0.5	10.41 ± 0.5	9.91 ± 0.6	9.42 ± 0.5
	***p*-value**		0.36	0.47	0.53	0.21	0.93	0.74	0.89	0.41	0.16	0.69	0.66	0.87	0.3	0.61	0.57	0.18
**TRU9l**	UU	18	50.50 ± 2.0	3325.00 ± 359	35.11 ± 5.0	17.32 ± 1.9	30.68 ± 1.9	27.70 ± 1.5	25.81 ± 1.3	2.90 ± 0.2	3.83 ± 0.5	15.30 ± 1.1	13.32 ± 0.8	13.37 ± 0.7	8.88 ± 0.5	10.35 ± 0.5	9.96 ± 0.6	9.34 ± 0.5
	Uu + uu	7	49.92 ± 1.7	3305.71 ± 379	34.42 ± 1.0	18.27 ± 2.0	31.74 ± 2.2	28.44 ± 2.5	26.25 ± 1.7	2.77 ± 0.3	3.97 ± 0.3	15.92 ± 1.3	13.98 ± 1.5	13.35 ± 0.7	9.07 ± 0.8	10.35 ± 0.9	9.98 ± 0.5	9.05 ± 0.7
	***p*-value**		0.51	0.9	0.73	0.29	0.26	0.38	0.51	0.36	0.54	0.25	0.18	0.96	0.52	0.99	0.92	0.19

If the Levene’s test for equality of variances *p* > 0.05 then equal variances assumed Sig (2-tailed) *p* values of *T*-Tests were given. If the Levene’s test for equality of variances *p* < 0.05 then equal variances not assumed Sig (2-tailed) *p* values of *T*-Tests were given. * The data and *p* values adjusted for maternal height (cm), BMI pre-pregnancy (kg/m^2^), BMI terminal (kg/m^2^), and weeks of gestation by one-way analysis of covariance (ANCOVA). ^†^ Corrected effect size was calculated with Hedge’s g (H*g*). ^***ϕ***^ Post-hoc power analysis. Abbreviations: VDR: Vitamin D receptor; 25(OH)D: 25-hydroxy-vitamin D. Digits in bold refer to significant effects.

**Table 4:** Birth neonatal anthropometry (neonatal vitamin D status at birth >25 nmol/L) according to neonatal VDR polymorphisms.

SNP	Genotype	N	Height (cm)	Weight (g)	Head Circ/ce (cm)	Neck Rump Length (cm)	Chest Circ/ce (cm)	Abdominal Circ/ce (cm)	Abdominal Circ/ce Iliac (cm)	Skin Fold Abdominal (cm)	Skin Fold High Anterior (cm)	High Thigh Circ/ce (cm)	Middle Thigh Circ/ce (cm)	Upper Arm Length (cm)	Lower Arm Radial Circ/ce (cm)	Lower Leg Calf Circ/ce (cm)	Femur Length (cm)	Knee-Heel Length (cm)
**APAl**	AA	18	50.50 ± 1.8	3241.66 ± 423	34.30 ± 0.8	17.80 ± 2.3	30.98 ± 2.0	28.15 ± 2.1	25.94 ± 2.0	2.93 ± 0.3	3.76 ± 0.4	15.27 ± 1.5	13.15 ± 1.2	13.77 ± 0.5	8.96 ± 0.8	10.16 ± 0.9	9.93 ± 0.6	9.18 ± 0.4
	Aa + aa	23	50.63 ± 2.1	3301.73 ± 475	33.91 ± 1.5	17.65 ± 2.3	30.88 ± 1.9	28.29 ± 2.1	25.93 ± 1.7	3.07 ± 0.7	3.82 ± 0.4	15.43 ± 1.7	13.38 ± 1.2	13.88 ± 1.3	9.04 ± 0.5	10.17 ± 0.9	9.93 ± 0.4	8.98 ± 0.7
	***p*-value**		0.84	0.67	0.33	0.84	0.87	0.84	0.99	0.43	0.65	0.75	0.55	0.75	0.69	0.96	0.99	0.33
**TAQl**	TT	17	51.35 ± 2.1	3423.52 ± 488	34.44 ± 0.9	17.69 ± 2.9	31.34 ± 1.9	28.83 ± 2.1	26.20 ± 1.8	3.09 ± 0.8	3.90 ± 0.4	15.68 ± 1.7	13.58 ± 1.2	14.10 ± 1.4	9.15 ± 0.6	10.41 ± 0.8	10.01 ± 0.5	9.05 ± 0.8
			(50.79 ± 2.1) *															
	Tt + tt	24	50.02 ± 1.7	3170.41 ± 395	33.83 ± 1.4	17.73 ± 1.7	30.64 ± 1.9	27.80 ± 2.0	25.74 ± 1.9	2.95 ± 0.3	3.72 ± 0.4	15.14 ± 1.5	13.06 ± 1.1	13.64 ± 0.5	8.90 ± 0.7	9.99 ± 0.9	9.87 ± 0.6	9.08 ± 0.5
			*(50.75* ± 2.1) *															
	***p*-value**		**0.035**	**0.075**	0.13	0.95	0.26	0.12	0.45	0.47	0.16	0.3	0.18	0.16	0.26	0.14	0.44	0.88
			0.88 *															
**BSMl**	BB	16	50.40 ± 1.9	3220.00 ± 420	34.28 ± 0.8	18.06 ± 1.8	30.91 ± 2.1	28.18 ± 2.2	26.08 ± 2.1	2.95 ± 0.3	3.75 ± 0.4	15.43 ± 1.5	13.26 ± 1.2	13.78 ± 0.5	9.01 ± 0.8	10.20 ± 0.9	9.91 ± 0.7	9.16 ± 0.5
	Bb + bb	25	50.68 ± 2.1	3310.80 ± 471	33.96 ± 1.5	17.49 ± 2.5	30.94 ± 1.8	28.26 ± 2.1	25.84 ± 1.7	3.05 ± 0.6	3.83 ± 0.3	15.32 ± 1.7	13.29 ± 1.2	13.86 ± 1.2	9.00 ± 0.5	10.14 ± 0.8	9.94 ± 0.4	9.00 ± 0.7
	***p*-value**		0.67	0.53	0.44	0.44	0.96	0.91	0.69	0.57	0.54	0.84	0.94	0.81	0.98	0.86	0.89	0.44
**FOKI**	FF	20	50.65 ± 2.2	3370.50 ± 453	33.92 ± 1.7	18.31 ± 2.3	31.31 ± 1.8	28.44 ± 2.1	26.32 ± 2.0	3.08 ± 0.8	3.90 ± 0.3	15.64 ± 1.8	13.44 ± 1.3	14.03 ± 1.2	9.17 ± 0.7	10.34 ± 1.0	9.97 ± 0.5	9.16 ± 0.5
	Ff + ff	21	50.50 ± 1.8	3184.76 ± 435	34.23 ± 0.6	17.15 ± 2.0	30.56 ± 1.9	28.03 ± 2.1	25.58 ± 1.6	2.95 ± 0.2	3.70 ± 0.4	15.10 ± 1.4	13.12 ± 1.0	13.64 ± 0.6	8.85 ± 0.5	10.00 ± 1.0	9.89 ± 0.6	8.98 ± 0.7
	***p*-value**		0.81	0.18	0.45	0.1	0.22	0.54	0.21	0.49	0.13	0.3	0.41	0.23	0.13	0.22	0.66	0.39
**TRU9l**	UU	20	50.22 ± 1.7	3187.50 ± 459	33.85 ± 1.6	17.31 ± 2.3	30.29 ± 1.7	27.44 ± 2.2	25.42 ± 1.7	3.11 ± 0.7	3.83 ± 0.4	14.77 ± 1.4	12.89 ± 1.1	13.80 ± 1.3	8.89 ± 0.6	9.91 ± 0.8	9.97 ± 0.4	9.04 ± 0.7
							(30.93 ± 2.3) *	(28.02 ± 2.9) *				(15.53 ± 1.8) *	(13.42 ± 1.5) *					
	Uu + uu	21	50.90 ± 2.1	3359.04 ± 433	34.30 ± 0.7	18.10 ± 2.1	31.53 ± 1.9	28.98 ± 1.7	26.43 ± 1.8	2.92 ± 0.3	3.77 ± 0.4	15.93 ± 1.6	13.65 ± 1.2	13.86 ± 0.7	9.11 ± 0.6	10.40 ± 0.8	9.89 ± 0.6	9.09 ± 0.5
							(31.05 ± 1.8) *	(28.39 ± 1.8) *				(15.24 ± 1.5) *	(13.30 ± 1.0) *					
	***p*-value**		0.28	0.22	0.25	0.26	**0.038**	**0.019**	**0.084**	0.31	0.65	**0.022**	**0.043**	0.85	0.29	**0.08**	0.66	0.8
							0.96 *	0.74 *				0.78 *	0.94 *					

If the Levene’s test for equality of variances *p* > 0.05 then equal variances assumed Sig (2-tailed) *p* values of *T*-Tests were given. If the Levene’s test for equality of variances *p* < 0.05 then equal variances not assumed Sig (2-tailed) *p* values of *T*-Tests were given. * The data and *p* values adjusted for maternal height (cm), BMI pre-pregnancy (kg/m^2^), BMI terminal (kg/m^2^), and weeks of gestation by one-way analysis of covariance (ANCOVA). Abbreviations: VDR: Vitamin D receptor; 25(OH)D: 25-hydroxy-vitamin D. Digits in bold refer to significant effects.

**Table 5:** Birth neonatal anthropometry (neonatal vitamin D status at birth >50 nmol/L) according to neonatal VDR polymorphisms.

SNP	Genotype	N	Height (cm)	Weight (g)	Head Circ/ce (cm)	Neck Rump Length (cm)	Chest Circ/ce (cm)	Abdominal Circ/ce (cm)	Abdominal Circ/ce Iliac (cm)	Skin fold Abdominal (cm)	Skin Fold Thigh Anterior (cm)	High Thigh Circ/ce (cm)	Middle Thigh Circ/ce (cm)	Upper Arm Length (cm)	Lower Arm Radial Circ/ce (cm)	Lower Leg Calf Circ/ce (cm)	Femur Length (cm)	Knee-Heel Length (cm)
**APAl**	AA	3	51.66 ± 1.4	3336.66 ± 457	35.16 ± 0.2	17.96 ± 1.9	17.96 ± 0.6	32.80 ± 1.0	26.40 ± 0.4	2.73 ± 0.4	3.86 ± 0.2	16.13 ± 0.5	13.90 ± 0.1	14.16 ± 0.4	8.76 ± 0.5	10.06 ± 0.8	8.86 ± 0.4	9.93 ± 1.0
	Aa + aa	11	50.36 ± 2.0	3223.63 ± 542	33.72 ± 1.8	18.06 ± 3.0	18.06 ± 2.0	31.47 ± 2.5	26.66 ± 2.2	2.96 ± 0.4	3.94 ± 0.4	15.80 ± 1.7	13.56 ± 1.3	14.22 ± 1.6	9.21 ± 0.8	10.38 ± 1.1	8.98 ± 0.4	9.83 ± 0.8
	***p*-value**		NA	NA	NA	NA	NA	NA	NA	NA	NA	NA	NA	NA	NA	NA	NA	NA
**TAQl**	TT	7	50.78 ± 1.7	3218.57 ± 608	34.21 ± 1.1	17.15 ± 2.9	31.47 ± 1.9	29.21 ± 2.4	26.52 ± 2.3	2.82 ± 0.3	3.97 ± 0.5	15.22 ± 1.7	13.30 ± 1.3	14.54 ± 1.9	9.11 ± 0.6	10.22 ± 1.0	9.85 ± 0.4	8.88 ± 1.0
	Tt + tt	7	50.50 ± 2.2	3277.14 ± 438	33.85 ± 2.2	18.92 ± 2.4	32.04 ± 1.9	28.54 ± 2.2	26.68 ± 1.8	3.00 ± 0.4	3.88 ± 0.3	16.52 ± 0.9	13.97 ± 1.1	13.88 ± 0.6	9.11 ± 0.9	10.40 ± 1.0	9.85 ± 0.4	9.02 ± 0.7
	***p*-value**		0.79	0.84	0.71	0.24	0.6	0.6	0.89	0.45	0.71	0.11	0.33	0.41	1	0.76	1	0.77
**BSMl**	BB	3	51.66 ± 1.5	3336.66 ± 457	35.16 ± 0.2	17.96 ± 1.9	32.80 ± 0.6	29.06 ± 1.06	26.40 ± 0.4	2.73 ± 0.4	3.86 ± 0.2	16.13 ± 0.5	13.90 ± 0.3	14.16 ± 0.45	8.76 ± 0.5	10.06 ± 0.8	9.93 ± 0.4	8.86 ± 1.0
	Bb + bb	11	50.36 ± 2.0	3223.63 ± 542	33.72 ± 1.8	18.06 ± 3.1	31.47 ± 2.0	28.82 ± 2.56	26.66 ± 2.3	2.96 ± 0.3	3.94 ± 0.4	15.81 ± 1.6	13.56 ± 1.3	14.22 ± 1.6	9.21 ± 0.8	10.38 ± 1.0	9.83 ± 0.4	8.98 ± 0.8
	***p*-value**		NA	NA	NA	NA	NA	NA	NA	NA	NA	NA	NA	NA	NA	NA	NA	NA
**FOKI**	FF	9	50.61 ± 2.3	3294.44 ± 442	33.72 ± 2.0	18.42 ± 1.9	31.54 ± 1.5	28.42 ± 2.4	26.63 ± 2.1	2.93 ± 0.4	4.00 ± 0.5	16.11 ± 1.1	13.66 ± 0.9	14.58 ± 1.6	9.12 ± 0.7	10.32 ± 0.9	9.90 ± 0.4	9.14 ± 0.3
	Ff + ff	5	50.70 ± 1.3	3164.00 ± 664	34.60 ± 0.8	17.36 ± 4.0	32.14 ± 2.8	29.70 ± 1.8	26.56 ± 2.0	2.88 ± 0.2	3.80 ± 0.3	15.46 ± 2.1	13.58 ± 1.8	13.54 ± 0.5	9.10 ± 0.9	10.30 ± 1.2	9.78 ± 0.4	8.62 ± 1.4
	***p*-value**		0.93	0.66	0.38	0.51	0.6	0.33	0.95	0.82	0.4	0.46	0.91	0.19	0.96	0.97	0.62	0.46
**TRU9l**	UU	10	50.60 ± 2.1	3228.00 ± 554	34.00 ± 1.9	18.63 ± 2.3	31.49 ± 2.1	28.42 ± 2.5	26.25 ± 2.1	3.00 ± 0.3	3.86 ± 0.4	15.78 ± 1.6	13.55 ± 1.3	14.31 ± 1.6	9.06 ± 0.8	10.18 ± 1.1	9.77 ± 0.4	8.88 ± 1.0
	Uu + uu	4	50.75 ± 1.8	3297.50 ± 449	34.12 ± 1.1	16.57 ± 3.5	32.42 ± 0.9	30.02 ± 0.7	27.50 ± 1.6	2.70 ± 0.4	4.10 ± 0.3	16.12 ± 1.3	13.85 ± 1.0	13.97 ± 0.3	9.25 ± 0.6	10.65 ± 0.7	10.07 ± 0.3	9.15 ± 0.2
	***p*-value**		NA	NA	NA	NA	NA	NA	NA	NA	NA	NA	NA	NA	NA	NA	NA	NA

If the Levene’s test for equality of variances *p* > 0.05 then equal variances assumed Sig (2-tailed) *p* values of *T*-Tests were given. If the Levene’s test for equality of variances *p* < 0.05 then equal variances not assumed Sig (2-tailed) *p* values of *T*-Tests were given. Abbreviations: VDR: Vitamin D receptor; 25(OH)D: 25-hydroxy-vitamin D, NA: Not applicable.

**Table 6:** Birth neonatal anthropometry (maternal vitamin D status at birth <50 nmol/L) according to maternal VDR polymorphisms.

SNP	Genotype	N	Height (cm)	Weight (g)	Head Circ/ce (cm)	Neck Rump Length (cm)	Chest Circ/ce (cm)	Abdominal Circ/ce (cm)	Abdominal Circ/ce Iliac (cm)	Skin Fold Abdominal (cm)	Skin Fold High Anterior (cm)	High Thigh Circ/ce (cm)	Middle Thigh Circ/ce (cm)	Upper Arm Length (cm)	Lower Arm Radial Circ/ce (cm)	Lower Leg Calf Circ/ce (cm)	Femur Length (cm)	Knee-Heel Length (cm)
**APAl**	AA	18	49.66 ± 1.6	3282.77 ± 375	34.41 ± 0.7	17.93 ± 1.7	30.26 ± 1.9	27.76 ± 2.0	25.50 ± 1.7	2.81 ± 0.3	3.60 ± 0.5	15.05 ± 1.2	13.16 ± 1.2	13.56 ± 0.5	8.87 ± 0.8	10.21 ± 0.7	9.96 ± 0.7	9.24 ± 0.4
											(3.64 ± 0.4) *							
	Aa + aa	25	50.54 ± 1.9	3320.40 ± 361	34.46 ± 4.4	17.43 ± 1.8	31.17 ± 1.8	28.08 ± 1.6	25.99 ± 1.5	3.08 ± 0.6	3.96 ± 0.4	15.55 ± 1.4	13.48 ± 0.9	13.41 ± 0.7	9.04 ± 0.6	10.30 ± 0.7	9.94 ± 0.5	9.24 ± 0.5
											(3.98 ± 0.4) *							
	***p*-value**		0.13	0.74	0.96	0.35	0.13	0.57	0.32	0.12	**0.017**	0.22	0.34	0.47	0.18	0.44	0.93	0.99
											0.097 *							
**TAQl**	TT	14	50.50 ± 2.2	3520.71 ± 426	35.42 ± 5.7	18.58 ± 1.5	31.99 ± 2.0	29.27 ± 1.8	26.84 ± 1.8	3.18 ± 0.8	4.04 ± 0.4	16.16 ± 1.4	14.07 ± 1.2	13.60 ± 0.6	9.35 ± 0.7	10.73 ± 0.8	10.07 ± 0.6	9.43 ± 0.6
				(3503.33 ± 348) *		(18.32 ± 1.1) *	(31.91 ± 1.4) *	(29.01 ± 1.3) *	(26.51 ± 1.6) *		(4.00 ± 0.4) *	(15.86 ± 1.2) *	(13.85 ± 1.0) *		(9.17 ± 0.6) *	(10.56 ± 0.7) *		
	Tt + tt	29	50.01 ± 1.6	3200.34 ± 281	33.96 ± 0.9	17.18 ± 1.7	30.21 ± 1.5	27.31 ± 1.4	25.27 ± 1.1	2.86 ± 0.2	3.69 ± 0.5	14.94 ± 1.1	12.99 ± 0.7	13.41 ± 0.6	8.78 ± 0.5	10.03 ± 0.6	9.89 ± 0.5	9.15 ± 0.3
				(3146.31 ± 218) *		(16.82 ± 1.7) *	(30.07 ± 1.3) *	(26.99 ± 1.3) *	(25.00 ± 0.8) *		(3.80 ± 0.4) *	(14.88 ± 1.0) *	(12.97 ± 0.7) *		(8.66 ± 0.3) *	(9.91 ± 0.4) *		
	***p*-value**		0.43	**0.019**	0.36	**0.012**	**0.003**	**0.001**	**0.001**	0.07	**0.031**	**0.003**	**0.001**	0.35	**0.018**	**0.003**	0.38	0.17
	***adjusted p***			0.001 *		0.036 *	0.004 *	0.001 *	0.001 *		0.15 *	0.028 *	0.008 *		0.002 *	0.020 *		
	***Effect size***			***1.341 ^†^***		***0.976 ^†^***	***1.381 ^†^***	***1.554 ^†^***	***1.351 ^†^***			***0.918 ^†^***	***1.090 ^†^***		***1.217 ^†^***	***1.263 ^†^***		
	***Power***			***0.94 ^ϕ^***		***0.94 ^ϕ^***	***0.99 ^ϕ^***	***1.00 ^ϕ^***	***0.92 ^ϕ^***			***0.75 ^ϕ^***	***0.84 ^ϕ^***		***0.85 ^ϕ^***	***0.90 ^ϕ^***		
**BSMl**	BB	16	49.53 ± 1.6	3202.50 ± 310	34.31 ± 0.7	17.58 ± 1.4	29.91 ± 1.5	27.45 ± 1.5	27.45 ± 1.5	2.78 ± 0.3	3.53 ± 0.5	14.93 ± 0.9	12.98 ± 0.7	13.54 ± 0.5	8.80 ± 0.6	10.13 ± 0.6	9.90 ± 0.7	9.17 ± 0.4
							(29.84 ± 1.0) *				(3.60 ± 0.4) *							
	Bb + bb	27	50.55 ± 1.8	3365.18 ± 384	34.51 ± 4.2	17.67 ± 1.9	31.30 ± 1.9	28.24 ± 1.9	28.24 ± 1.9	3.07 ± 0.6	3.97 ± 0.4	15.58 ± 1.4	13.56 ± 1.1	13.43 ± 0.6	9.06 ± 0.6	10.34 ± 0.8	9.98 ± 0.5	9.28 ± 0.5
							(31.05 ± 1.7) *				(3.98 ± 0.4) *							
	***p*-value**		0.081	0.15	0.84	0.87	**0.021**	0.17	0.17	0.1	**0.004**	0.11	0.082	0.6	0.21	0.37	0.68	0.5
	***adjusted p***						0.079 *				0.041 *							
	***Effect size***										***0.950 ^†^***							
	***Power***										***0.85 ^ϕ^***							
**FOKI**	FF	15	49.66 ± 1.6	3373.33 ± 348	33.86 ± 1.2	18.52 ± 1.4	30.78 ± 1.7	28.05 ± 2.0	25.89 ± 1.7	3.01 ± 0.8	3.82 ± 0.5	15.24 ± 1.5	15.24 ± 1.5	13.46 ± 0.5	8.98 ± 0.8	10.30 ± 0.8	10.08 ± 0.5	9.16 ± 0.5
						(18.57 ± 1.1) *												
	Ff + ff	28	50.44 ± 1.9	3267.85 ± 371	34.75 ± 4.0	17.17 ± 1.7	30.79 ± 2.0	27.89 ± 1.7	25.73 ± 1.5	2.94 ± 0.2	3.80 ± 0.5	15.39 ± 1.2	15.39 ± 0.2	13.48 ± 0.6	8.96 ± 0.5	10.24 ± 0.6	9.87 ± 0.6	9.28 ± 0.5
						(16.79 ± 1.6) *												
	***p*-value**		0.19	0.37	0.41	**0.014**	0.99	0.79	0.75	0.69	0.86	0.72	0.78	0.94	0.91	0.79	0.3	0.43
	***adjusted p***					0.042 *												
	***Effect size***					***1.228 ^†^***												
	***Power***					***0.99 ^ϕ^***												
**TRU9l**	UU	26	50.44 ± 1.8	3286.92 ± 368	34.67 ± 4.2	17.28 ± 1.7	30.48 ± 1.7	27.46 ± 1.5	25.58 ± 1.4	2.95 ± 0.3	3.86 ± 0.5	15.05 ± 1.1	12.98 ± 1.3	13.40 ± 0.7	8.88 ± 0.5	10.17 ± 0.7	10.04 ± 0.5	9.33 ± 0.4
								(27.21 ± 1.5) *										
	Uu + uu	17	49.76 ± 1.8	3331.76 ± 365	34.08 ± 0.8	18.19 ± 1.5	31.26 ± 2.1	28.70 ± 2.1	26.10 ± 1.7	2.92 ± 0.4	3.72 ± 0.4	15.78 ± 1.5	13.86 ± 1.3	13.58 ± 0.5	9.10 ± 0.7	10.40 ± 0.8	9.80 ± 0.6	9.10 ± 0.5
								(28.30 ± 1.5) *										
	***p*-value**		0.24	0.69	0.58	0.093	0.19	**0.031**	0.28	0.31	0.4	0.078	0.12	0.38	0.27	0.34	0.22	0.15
								0.083 *										

If the Levene’s test for equality of variances *p* > 0.05 then equal variances assumed Sig (2-tailed) *p* values of *T*-Tests were given. If the Levene’s test for equality of variances *p* < 0.05 then equal variances not assumed Sig (2-tailed) *p* values of *T*-Tests were given. * The data and *p* values adjusted for maternal height (cm), BMI pre-pregnancy (kg/m^2^), BMI terminal (kg/m^2^) and weeks of gestation by one-way analysis of covariance (ANCOVA). ^†^ Corrected effect size was calculated with Hedge’s g (H*g*). ***^ϕ^*** Post-hoc power analysis. Abbreviations: VDR: Vitamin D receptor; 25(OH)D: 25-hydroxy-vitamin D. Digits in bold refer to significant effects.

**Table 7:** Birth neonatal anthropometry (maternal vitamin D status at birth >50 nmol/L) according to maternal VDR polymorphisms.

SNP	Genotype	N	Height (cm)	Weight (g)	Head Circ/ce (cm)	Neck Rump Length (cm)	Chest Circ/ce (cm)	Abdominal Circ/ce (cm)	Abdominal Circ/ce Iliac (cm)	Skin Fold Abdominal (cm)	Skin Fold High Anterior (cm)	High Thigh Circ/ce (cm)	Middle Thigh Circ/ce (cm)	Upper Arm Length (cm)	Lower Arm Radial Circ/ce (cm)	Lower Leg Calf Circ/ce (cm)	Femur Length (cm)	Knee-Heel Length (cm)
**APAl**	AA	9	51.16 ± 1.8	3178.88 ± 437	34.44 ± 0.8	17.55 ± 3.1	32.03 ± 2.1	28.74 ± 2.3	26.67 ± 2.2	2.95 ± 0.4	3.97 ± 0.2	15.70 ± 1.7	13.41 ± 1.5	25.93 ± 0.8	9.06 ± 0.8	10.23 ± 1.1	9.96 ± 0.4	9.27 ± 0.4
	Aa + aa	14	51.00 ± 2.2	3326.42 ± 550	34.25 ± 1.7	17.82 ± 2.8	30.74 ± 1.8	28.18 ± 2.4	25.93 ± 1.8	2.92 ± 0.3	3.78 ± 0.3	15.42 ± 1.8	13.40 ± 1.3	13.87 ± 0.6	8.97 ± 0.6	10.16 ± 0.9	9.92 ± 0.4	8.81 ± 0.9
	***p*-value**		0.85	0.5	0.75	0.82	0.14	0.59	0.39	0.87	0.19	0.72	0.98	0.72	0.75	0.87	0.82	0.17
**TAQl**	TT	11	51.90 ± 1.9	3372.72 ± 580	34.72 ± 0.9	17.47 ± 3.5	31.09 ± 1.9	28.46 ± 2.5	26.01 ± 1.9	2.92 ± 0.4	3.85 ± 0.4	15.53 ± 1.7	13.48 ± 1.3	14.30 ± 1.7	9.08 ± 0.6	10.26 ± 0.9	9.98 ± 0.5	8.88 ± 0.9
	Tt + tt	12	50.29 ± 1.9	3173.33 ± 425	33.95 ± 1.7	17.95 ± 2.1	31.39 ± 2.2	28.35 ± 2.2	26.41 ± 2.18	2.95 ± 0.3	3.86 ± 0.3	15.53 ± 1.8	13.33 ± 1.4	13.71 ± 0.6	8.94 ± 0.8	10.12 ± 1.1	9.90 ± 0.4	9.10 ± 0.6
	***p*-value**		**0.06**	0.35	0.21	0.69	0.73	0.91	0.64	0.89	0.93	0.99	0.8	0.28	0.64	0.74	0.69	0.52
**BSMl**	BB	15	51.06 ± 1.8	3200.00 ± 462	34.50 ± 0.8	18.28 ± 2.3	31.93 ± 2.3	28.70 ± 2.4	26.72 ± 2.4	2.97 ± 0.4	4.00 ± 0.2	15.87 ± 1.8	13.53 ± 1.6	13.88 ± 0.7	9.07 ± 0.9	10.23 ± 1.2	10.00 ± 0.4	9.27 ± 0.4
	Bb + bb	8	51.06 ± 2.2	3305.33 ± 536	34.23 ± 1.6	17.42 ± 3.1	30.88 ± 1.9	28.24 ± 2.3	25.96 ± 1.7	2.92 ± 0.3	3.78 ± 0.4	15.35 ± 1.7	13.33 ± 1.3	14.06 ± 1.5	8.97 ± 0.6	10.16 ± 0.9	9.90 ± 0.4	8.84 ± 0.8
	***p*-value**		0.99	0.64	0.68	0.5	0.25	0.67	0.39	0.75	0.15	0.51	0.74	0.77	0.75	0.87	0.66	0.22
**FOKI**	FF	15	51.13 ± 2.1	3291.33 ± 470	34.20 ± 1.6	18.03 ± 2.7	31.59 ± 2.0	28.36 ± 2.4	26.46 ± 2.1	2.96 ± 0.4	3.84 ± 0.3	15.84 ± 1.7	13.60 ± 1.4	14.14 ± 1.4	9.10 ± 0.7	10.33 ± 1.1	9.91 ± 0.4	9.12 ± 0.5
	Ff + ff	8	50.93 ± 2.0	3226.25 ± 593	34.56 ± 0.8	17.13 ± 3.2	30.60 ± 2.0	28.47 ± 2.4	25.78 ± 1.8	2.90 ± 0.3	3.90 ± 0.4	14.95 ± 1.7	13.03 ± 1.3	13.73 ± 0.9	8.83 ± 0.6	9.92 ± 0.8	9.98 ± 0.6	8.75 ± 1.0
	***p*-value**		0.83	0.77	0.57	0.48	0.28	0.91	0.45	0.72	0.69	0.25	0.36	0.49	0.39	0.35	0.73	0.28
**TRU9l**	UU	12	50.16 ± 1.9	3178.33 ± 512	33.95 ± 1.8	17.39 ± 2.9	30.47 ± 2.0	27.78 ± 2.6	25.67 ± 1.9	2.95 ± 0.3	3.76 ± 0.4	14.95 ± 1.7	12.98 ± 1.3	14.01 ± 1.5	8.91 ± 0.7	10.01 ± 0.9	9.80 ± 0.3	8.94 ± 0.9
			(50.50 ± 1.5) *															
	Uu + uu	11	52.04 ± 1.7	3367.27 ± 499	34.72 ± 0.6	18.08 ± 2.8	32.09 ± 1.7	29.08 ± 1.8	26.82 ± 1.9	2.92 ± 0.4	3.96 ± 0.2	16.16 ± 1.8	13.86 ± 1.3	13.98 ± 0.9	9.11 ± 0.7	10.39 ± 1.0	10.09 ± 0.5	9.05 ± 0.5
			(49.72 ± 2.0) *															
	***p*-value**		**0.026**	0.38	0.21	0.57	**0.059**	0.19	0.17	0.89	0.17	0.1	0.13	0.95	0.52	0.36	0.14	0.74
	***adjusted p***		0.032 *															
	***Effect size***		***0.444 ^†^***															
	***Power***		***0.18 ^ϕ^***															

If the Levene’s test for equality of variances *p* > 0.05 then equal variances assumed Sig (2-tailed) *p* values of *T*-Tests were given. If the Levene’s test for equality of variances *p* < 0.05 then equal variances not assumed Sig (2-tailed) *p* values of *T*-Tests were given. * The data and *p* values adjusted for maternal height (cm), BMI pre-pregnancy (kg/m^2^), BMI terminal (kg/m^2^), and weeks of gestation by one-way analysis of covariance (ANCOVA). ^†^ Corrected effect size was calculated with Hedge’s g (H*g*). ***^ϕ^*** Post-hoc power analysis. Abbreviations: VDR: Vitamin D receptor; 25(OH)D: 25-hydroxy-vitamin D. Digits in bold refer to significant effects.

**Table 8:** Birth neonatal anthropometry (maternal vitamin D status at birth <75 nmol/L) according to neonatal VDR polymorphisms.

SNP	Genotype	N	Height (cm)	Weight (g)	Head Circ/ce (cm)	Neck Rump Length (cm)	Chest Circ/ce (cm)	Abdominal Circ/ce (cm)	Abdominal Circ/ce Iliac (cm)	Skin Fold Abdominal (cm)	Skin Fold High Anterior (cm)	High Thigh Circ/ce (cm)	Middle Thigh Circ/ce (cm)	Upper Arm Length (cm)	Lower Arm Radial Circ/ce (cm)	Lower Leg Calf Circ/ce (cm)	Femur Length (cm)	Knee-Heel Length (cm)
**APAl**	AA	18	50.58 ± 2.1	3251.66 ± 407	35.72 ± 4.8	17.30 ± 2.0	30.54 ± 1.7	27.71 ± 1.4	25.43 ± 1.5	2.80 ± 0.2	3.81 ± 0.4	14.80 ± 1.0	13.03 ± 0.9	13.58 ± 0.7	8.69 ± 0.5	9.95 ± 0.7	9.91 ± 0.7	9.21 ± 0.5
					(34.50 ± 0.5) *							(14.97 ± 0.8) *			(8.56 ± 0.2) *			
	Aa + aa	37	50.39 ± 1.9	3334.05 ± 397	33.89 ± 1.1	17.97 ± 2.0	31.18 ± 2.0	28.32 ± 2.1	26.15 ± 1.6	3.03 ± 0.6	3.87 ± 0.4	15.70 ± 1.8	13.57 ± 1.2	13.71 ± 1.1	9.09 ± 0.6	10.37 ± 0.8	9.94 ± 0.5	9.18 ± 0.5
					(34.00 ± 0.9) *							(15.64 ± 1.5) *			(9.05 ± 0.6) *			
	***p*-value**		0.74	0.47	**0.032**	0.24	0.25	0.27	0.12	0.12	0.66	**0.029**	0.1	0.67	**0.031**	0.067	0.88	0.81
	***adjusted p***				0.24 *							0.20 *			0.043 *			
	***Effect size***														***0.966 ^†^***			
	***Power***														***0.99 ^ϕ^***			
**TAQl**	TT	21	50.54 ± 1.8	3296.66 ± 405	34.11 ± 0.8	17.55 ± 2.1	31.27 ± 2.0	28.46 ± 2.3	28.46 ± 2.3	3.00 ± 0.7	3.91 ± 0.3	15.51 ± 1.5	13.62 ± 1.3	13.60 ± 1.3	9.02 ± 0.6	10.44 ± 0.7	9.85 ± 0.4	9.10 ± 0.5
	Tt + tt	34	50.39 ± 1.1	3313.52 ± 400	34.72 ± 3.7	17.88 ± 1.9	30.78 ± 1.8	27.91 ± 1.5	27.91 ± 1.5	2.92 ± 0.3	3.81 ± 0.5	15.34 ± 1.3	1.26 ± 1.0	13.70 ± 0.7	8.92 ± 0.6	10.11 ± 0.8	9.98 ± 0.6	9.24 ± 0.3
	***p*-value**		0.78	0.88	0.47	0.56	0.37	0.3	0.39	0.56	0.43	0.66	0.26	0.72	0.59	0.14	0.43	0.33
**BSMl**	BB	16	50.71 ± 2.2	3275.62 ± 426	35.93 ± 5.1	17.26 ± 2.1	30.53 ± 1.8	27.62 ± 1.5	25.38 ± 1.5	2.82 ± 0.2	3.83 ± 0.4	14.80 ± 1.1	13.01 ± 0.9	13.68 ± 0.7	8.71 ± 0.5	9.94 ± 0.8	9.94 ± 0.7	9.25 ± 0.5
												(15.02 ± 0.9) *						
	Bb + bb	39	50.34 ± 1.8	3320.00 ± 391	33.89 ± 1.0	17.95 ± 1.9	31.15 ± 1.7	28.33 ± 2.0	26.14 ± 1.5	3.01 ± 0.5	3.35 ± 0.4	15.65 ± 1.5	13.55 ± 1.2	13.66 ± 1.1	9.06 ± 0.6	10.35 ± 0.7	9.92 ± 0.5	9.16 ± 0.5
												(15.58 ± 1.5) *						
	***p*-value**		0.53	0.71	0.13	0.25	0.29	0.21	0.11	0.24	0.89	**0.048**	0.11	0.96	0.074	0.087	0.93	0.6
												0.25 *						
**FOKI**	FF	27	50.38 ± 2.2	3426.66 ± 459	34.92 ± 4.1	18.48 ± 1.6	31.15 ± 2.0	28.29 ± 2.2	26.15 ± 1.8	3.02 ± 0.6	3.85 ± 0.4	15.52 ± 1.5	13.58 ± 1.2	13.85 ± 1.2	9.07 ± 0.7	10.32 ± 0.9	9.96 ± 0.5	9.12 ± 0.5
						(18.52 ± 1.8) *												
				(3311.42 ± 479) *														
	Ff + ff	28	50.51 ± 1.7	3191.77 ± 293	34.07 ± 0.9	17.05 ± 2.1	30.79 ± 1.8	27.96 ± 1.6	25.69 ± 1.3	2.88 ± 0.2	3.85 ± 0.4	15.29 ± 1.3	13.21 ± 1.0	13.48 ± 0.7	8.86 ± 0.5	10.15 ± 0.6	9.90 ± 0.5	9.25 ± 0.4
				(3250.00 ± 297) *		(17.04 ± 2.3) *												
	***p*-value**		0.81	**0.03**	0.29	**0.008**	0.49	0.52	0.3	0.31	0.98	0.56	0.23	0.17	0.24	0.46	0.67	0.35
				0.79 *		0.14 *												
**TRU9l**	UU	35	50.67 ± 2.1	3325.14 ± 400	34.65 ± 3.7	17.95 ± 1.9	30.95 ± 1.8	27.96 ± 1.8	25.98 ± 1.6	3.04 ± 0.5	3.88 ± 0.5	15.35 ± 1.3	13.33 ± 1.0	13.76 ± 1.2	9.00 ± 0.6	10.25 ± 0.8	10.02 ± 0.5	9.28 ± 0.4
	Uu + uu	20	50.07 ± 1.8	3275.50 ± 404	34.20 ± 0.7	17.49 ± 2.1	31.00 ± 2.0	28.41 ± 2.0	25.81 ± 1.6	2.80 ± 0.3	3.80 ± 0.3	15.51 ± 1.6	13.52 ± 1.3	13.50 ± 0.4	8.90 ± 0.6	10.21 ± 0.8	9.77 ± 0.6	9.03 ± 0.5
	***p*-value**		0.29	0.66	0.59	0.47	0.92	0.4	0.7	0.097	0.54	0.7	0.56	0.26	0.58	0.87	0.12	0.08

If the Levene’s test for equality of variances *p* > 0.05 then Equal variances assumed Sig (2-tailed) *p* values of *T*-Tests were given. If the Levene’s test for equality of variances *p* < 0.05 then equal variances not assumed Sig (2-tailed) *p* values of *T*-Tests were given. * The data and *p* values adjusted for maternal height (cm), BMI pre-pregnancy (kg/m^2^), BMI terminal (kg/m^2^), and weeks of gestation by one-way analysis of covariance (ANCOVA). ^†^ Corrected effect size was calculated with Hedge’s g (H*g*). ***^ϕ^*** Post-hoc power analysis. Abbreviations: VDR: Vitamin D receptor; 25(OH)D: 25-hydroxy-vitamin D. Digits in bold refer to significant effects.
